# An unusual case of extragenital primary syphilis^⋆^

**DOI:** 10.1016/j.abd.2022.09.018

**Published:** 2023-12-06

**Authors:** Miguel Santos-Coelho, Joana Alves Barbosa, Margarida Moura Valejo Coelho, Alexandre João

**Affiliations:** Service of Dermatovenereology, Hospital de Santo António dos Capuchos, Centro Hospitalar Universitário de Lisboa Central, Lisbon, Portugal

Dear Editor,

A twenty-eight year old man with no relevante personal history, sought a Dermatology consultation due to the appearance of an asymptomatic lesion on the right hand. The clinical history revealed the appearance of a pinkish papule, two months before, with progressive growth and subsequent ulceration, with no history of local trauma or similar lesions in the past.

The objective examination identified a painless ulcer, measuring 5 × 5 mm, on the dorsal side of the third finger of the right hand, with a bright red base and raised pinkish infiltrated borders (Fig. 1). No local adenopathies were identified and the remaining clinical examination showed no alterations.

**Figure 1:**
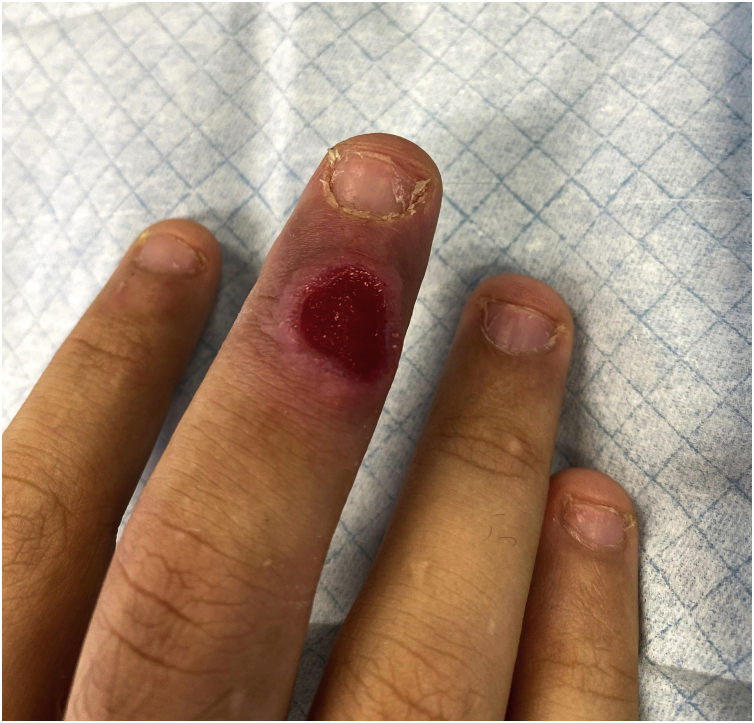
Ulcer on the third finger of the right hand.

Histopathology of a punch biopsy on the lesion border revealed a dense perivascular and interstitial lymphoplasmacytic infiltrate; immunohistochemical evaluation with anti-treponemal antibody staining showed massive epidermal, adnexal and vascular spirochete infiltration (Fig. 2). Laboratory assessment disclosed a positive FTA-ABS test and a VDRL title of 1/64. Serologies for the remaining sexually transmitted infections (HIV, hepatitis B and hepatitis C) were negative.

**Figure 2:**
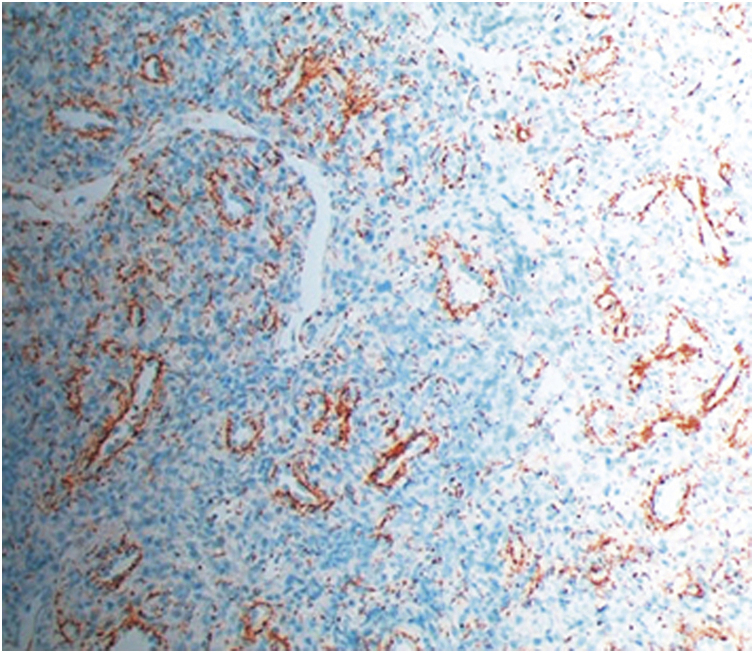
Evaluation by immunohistochemistry with anti-treponemal antiserum in the skin sample (×200).

A diagnosis of primary extragenital syphilis was established and the patient was medicated with intramuscular benzathine penicillin G (2.4 million IU), in a single dose, with complete resolution of the lesion in the following weeks.

## Discussion

Syphilis is an infection caused by the spirochete *Treponema pallidum* subsp. *pallidum*. The main method of transmission involves skin or mucous membrane contact with an infectious lesion, usually through sexual contact. The remaining cases correspond mostly to vertical transmission of the disease.^1^

Primary syphilis manifests through one or more asymptomatic ulcers at the site of inoculation, with characteristics similar to those described in the clinical case presented here, usually accompanied by local adenopathies. The lesion appears after a mean incubation period of three weeks (10–90 days), and the most common site for its development is the anogenital region.^2^ In the absence of treatment, healing occurs after a few weeks and about one-third of the patients develop manifestations of secondary syphilis later on.^2^

Extragenital primary syphilis is a rare event, corresponding to 2%–7% of reported cases,^3^ with the oral cavity being the most frequently affected area.^2^, ^4^ The natural history, treatment, and prognosis of primary lesions do not depend on their location.

The clinical differential diagnosis of extragenital lesions is extensive and should be guided by the clinical history. Cutaneous mycobacteriosis, herpetic infections, cutaneous leishmaniasis, and squamous cell carcinoma are conditions that must be taken in consideration.

The diagnosis is often made through a combination of clinical history, physical examination, and treponemal and non-treponemal serological tests. In this case, a skin biopsy was performed due to the atypical location of the lesion to exclude other etiologies. Direct darkfield examination and polymerase chain reaction (PCR) assessment are other diagnostic methods used sometimes.^2^

This clinical case illustrates a rare presentation of primary syphilis and demonstrates the importance of this differential diagnosis in lesions with the aforementioned characteristics, regardless of their location.

## Financial support

None declared.

## Authors' contributions

Miguel Santos Coelho: Data survey, data analysis and interpretation; drafting and editing of the manuscript; collection, analysis, and interpretation of data; intellectual participation in the propaedeutic and/or therapeutic conduct of the studied cases; critical review of the literature; approval of the final version of the manuscript.

Joana Alves Barbosa: Data survey, data analysis and interpretation; drafting and editing of the manuscript; collection, analysis, and interpretation of data; intellectual participation in the propaedeutic and/or therapeutic conduct of the studied cases; critical review of the literature; approval of the final version of the manuscript.

Margarida Moura Valejo Coelho: Critical review of the literature; approval of the final version of the manuscript.

Alexandre João: Data survey, data analysis and interpretation; collection, analysis, and interpretation of data; intellectual participation in the propaedeutic and/or therapeutic conduct of the studied cases; approval of the final version of the manuscript.

## Conflicts of interest

None declared.
